# A randomized controlled trial of a multicomponent online stress reduction intervention in inflammatory bowel disease

**DOI:** 10.1177/17562848221127238

**Published:** 2022-09-27

**Authors:** Farhad Peerani, Makayla Watt, Kathleen P Ismond, Reid Whitlock, Lindsy Ambrosio, Naomi Hotte, Nicholas Mitchell, Robert J Bailey, Karen Kroeker, Levinus A Dieleman, Jesse Siffledeen, Allen Lim, Karen Wong, Brendan P Halloran, Daniel C Baumgart, Lorian Taylor, Maitreyi Raman, Karen L Madsen, Puneeta Tandon

**Affiliations:** Division of Gastroenterology, Department of Medicine, University of Alberta, Edmonton, AB, Canada; Division of Gastroenterology, Department of Medicine, University of Alberta, Edmonton, AB, Canada; Division of Gastroenterology, Department of Medicine, University of Alberta, Edmonton, AB, Canada; Chronic Disease Innovation Centre, Seven Oaks General Hospital, Winnipeg, MB, Canada; Division of Gastroenterology, Department of Medicine, University of Alberta, Edmonton, AB, Canada; Division of Gastroenterology, Department of Medicine, University of Alberta, Edmonton, AB, Canada; Department of Psychiatry, University of Alberta, Edmonton, AB, Canada; Division of Gastroenterology, Department of Medicine, University of Alberta, Edmonton, AB, Canada; Division of Gastroenterology, Department of Medicine, University of Alberta, Edmonton, AB, Canada; Division of Gastroenterology, Department of Medicine, University of Alberta, Edmonton, AB, Canada; Division of Gastroenterology, Department of Medicine, University of Alberta, Edmonton, AB, Canada; Division of Gastroenterology, Department of Medicine, University of Alberta, Edmonton, AB, Canada; Division of Gastroenterology, Department of Medicine, University of Alberta, Edmonton, AB, Canada; Division of Gastroenterology, Department of Medicine, University of Alberta, Edmonton, AB, Canada; Division of Gastroenterology, Department of Medicine, University of Alberta, Edmonton, AB, Canada; Division of Gastroenterology, University of Calgary, Calgary, AB, Canada; Division of Gastroenterology, University of Calgary, Calgary, AB, Canada; Division of Gastroenterology, Department of Medicine, University of Alberta, Edmonton, AB, Canada; Division of Gastroenterology, Department of Medicine, University of Alberta, 130 University Campus NW, Edmonton, AB T6G 2X8, Canada

**Keywords:** inflammatory bowel disease, online intervention, stress reduction

## Abstract

**Background:**

Psychological stress negatively impacts inflammatory bowel disease (IBD) outcomes. Patients have prioritized access to online interventions; yet, the data on these have been limited by mixed in-person/online interventions, low adherence, and non-randomized controlled trial (RCT) design.

**Objectives:**

We assessed the efficacy of and adherence to a 12-week online multicomponent stress reduction intervention in IBD.

**Design:**

This is a RCT.

**Methods:**

Adult participants on stable IBD medical therapy with elevated stress levels from four centers were randomized to intervention or control groups. Intervention participants received a 12-week online program including a weekly yoga, breathwork and meditation video (target 2–3 times/week), a weekly cognitive behavioral therapy/positive psychology informed video activity, and weekly 10-min check-ins by a study team member. Control participants received weekly motivational messages by email. All patients received standard of care IBD therapy. The primary outcome was Cohen’s Perceived Stress Scale (PSS). Secondary outcomes evaluated mental health, resilience, health-related quality of life (HRQoL), symptom indices, acceptability, adherence, and inflammatory biomarkers. Analysis of covariance was used to determine between-group differences.

**Results:**

Of 150 screened patients, 101 were randomized to the intervention (*n* = 49) and control (*n* = 52) groups (mean age: 42.5 ± 14.1 years; M:F 1:3, 48% with ulcerative colitis and 52% with Crohn’s disease). The between-group PSS improved by 22.4% (95% confidence interval, 10.5–34.3, *p* < 0.001). Significant improvements were seen in mental health, resilience, and HRQoL measures, with a median satisfaction score of 89/100 at the end of the 12 weeks. In the 44/49 patients who completed the intervention, 91% achieved program adherence targets.

**Conclusion:**

This 12-week online intervention improved perceived stress, mental health, and HRQoL, but did not impact IBD symptom indices or inflammatory biomarkers. The program was readily adopted and adhered to by participants with high retention rates. After iterative refinement based on participant feedback, future studies will evaluate the impact of a longer/more intense intervention on disease course.

**Registration:**

ClinicalTrials.gov Identifier NCT03831750

**Plain Language Summary:**

**An online stress reduction intervention in inflammatory bowel disease patients improves stress, mental health, and quality of life**

People with inflammatory bowel disease (IBD) have high levels of stress, anxiety, and depression. Although IBD patients have expressed the need for online mental wellness interventions, the existing data to support these interventions in IBD are limited. In this trial, 101 IBD patients had the chance to participate in a 12-week online stress reduction intervention. In those patients randomly selected to participate in the online intervention, each week they received the following: a 20- to 30-min yoga, breathwork, and meditation video that they were asked to do 2–3 times a week, a 10- to 20-min mental wellness activity they were asked to do once during the week, and a 10-min telephone check-in with a study team member. Participants who were not selected to use the online intervention received a weekly motivational message by email. In all, 90 of the 101 participants (89%) completed the study with the mean age of participants being 43 years and the majority being females (75%). Ninety-one percent of participants who completed the intervention met the program target of doing the yoga, breathwork, and meditation video at least 2 times per week. Significant improvements were seen in perceived stress (by 22.4%), depression (by 29.5%), anxiety (by 23.7%), resilience (by 10.6%), and quality of life (by 8.9%). No changes were seen in IBD severity or in blood markers of inflammation. In conclusion, this study demonstrates evidence that a 12-week online stress reduction intervention had low dropout rates, high adherence and beneficial effects on stress, mental health, and quality of life measures. Continued feedback will be sought from study participants and our IBD patient partners to refine the intervention and assess the impact in future studies of patients with active IBD, as well as the impact of a longer/more intense intervention.

## Introduction

Inflammatory bowel disease (IBD) is a group of chronic, systemic inflammatory disorders with a high prevalence in North America and Europe.^1^ Although the underlying etiology is unclear, it is thought that IBD manifests due to a combination of environmental, immune, and genetic factors.^2,3^ One in five people with IBD has depression or anxiety, rates higher than expected in the general population.^4^ Psychiatric comorbidities predict increased healthcare utilization, decreased response to therapy, and worse health-related quality of life (HRQoL).^5,6^ Psychological stress is associated with symptomatic IBD flares, systemic pro-inflammatory responses, an earlier time to relapse, worse HRQoL, and adverse health outcomes.^7–11^ A recent study of 92 patients with ulcerative colitis (UC) and 137 patients with Crohn’s disease (CD) concluded that high levels of stress resilience were independently associated with a better HRQoL, lower disease activity, and fewer surgeries.^12^ In total, 11 national practice guidelines recommend the integration of stress reduction approaches in IBD management, yet this is not routinely provided to patients.^13^

Studies exploring psychological stress interventions in IBD have demonstrated mixed results,^14–23^ with some evidence that a greater effect may be seen in patients with higher baseline stress levels.^24,25^ To date, most programs have involved supervised in-person interventions. This requirement for travel is often a challenge for patients in rural areas or those who work or have caregiver responsibilities. This can lead to high dropout and low adherence rates.^18,20,22^ It is not surprising that more patients with IBD prefer online *versus* in-person stress reduction interventions, the appeal potentially increasing even further with the COVID-19 experience.^26^ There is limited randomized controlled trial (RCT) data to support purely online mental wellness interventions in IBD, with most online studies using a combination of in-person and online programming and reporting poor adherence rates.^15,27–29^

To build upon existing evidence, we worked collaboratively with our IBD patient partners to develop a 12-week online intervention [the Peace Power Pack (PPP)]. The intervention is unique in that it is fully online, multicomponent, and progressive over 12 weeks. It combines eastern stress reduction practices (i.e. yoga, breathwork, meditation) alongside western practices [i.e. cognitive behavioral therapy (CBT) and positive psychology informed activities]. Using a RCT design, our primary outcome was the change in Cohen’s Perceived Stress Scale (PSS).^30^ Our secondary outcomes included changes in psychological well-being, resiliency, HRQoL, IBD symptom indices, acceptability, and adherence. Exploratory analysis included effects on inflammatory biomarkers and serum cytokines. Our hypothesis was that the 12-week intervention delivered to patients with elevated PSS scores would positively impact primary and secondary outcomes when compared to the control group, be highly acceptable to participants and be associated with high adherence.

## Methods

### Study population

Eligible participants (⩾18 years of age) required an endoscopically and histologically confirmed diagnosis of either UC or CD, stable IBD therapy for 1 month prior to randomization, and an elevated PSS (⩾ 8). Stable therapy was defined as (1) no change in the dose of 5-aminosalicylate or immunomodulator; (2) no change in the dose or frequency of biologic; and (3) maintenance phase of biologic therapy. Exclusion criteria are as follows: Hospital Anxiety and Depression Scale (HADS) depression subcomponent score >10 (high probability of severe depression),^31^ PSS score < 7 [cutoff represents patients in the bottom quartile of PSS scores across a registry of IBD patients (*n* = 91) derived from the Inflammation, Microbiome, and Alimentation: Gastrointestinal and Neuropsychiatric Effects (IMAGINE) cohort^32^], *Clostridioides difficile* diagnosed within 1 month of baseline, steroid use within 1 month of baseline, new onset of treatment for anxiety or depression within the past 3 months, psychosis, or inability to provide informed written consent in English. To increase generalizability to a real-world population, a pre-existing diagnosis of depression or anxiety was not considered an exclusion criterion. Moreover, although stable IBD therapy for 1 month prior to randomization was required, patients were not excluded based on their clinical scores [Harvey-Bradshaw Index (HBI) for CD,^33^ partial Mayo (pMayo) score for UC^34^] or biochemical markers. Inclusion in the study did not prohibit standard of care IBD therapy, including escalation of medications for clinical or biochemical disease activity at the discretion of the participant’s primary gastroenterologist.

### Study design

The PPP RCT enrolled consecutive, consenting patients at four gastroenterology tertiary care centers in Edmonton, Alberta between February 2019 and March 2020. The UC and CD protocols were approved by the University of Alberta’s Research Ethics Office located in Edmonton, Alberta, Canada on 15 April 2018 (Pro00079377) and 26 June 2018 (Pro00082125), respectively. This trial was registered with www.ClinicalTrials.gov (Identifier NCT03831750). The study was advertised using clinic posters and recruitment through local IBD physicians. Patients were randomly allocated 1:1 to the intervention or control group using a computer randomization plan (www.randomization.com) and creation of securely stored sealed envelopes by personnel not involved in the RCT. Control group participants were given the option of taking part in the intervention after their 12-week control period was complete. This RCT was reported according to the CONSORT guidelines and the checklist is available as supplemental material.^35^

### Control group

To minimize engagement bias, standard of care participants received weekly themed emails containing a different one line motivational quote each week (e.g. Don’t believe everything you think, Jenny Bogart) and a countdown to the program start. All also received standard of care conventional IBD treatment, as per their treating gastroenterologist.

### Intervention group

In addition to standard of care conventional IBD treatment, intervention participants received the multicomponent stress reduction intervention (see more details in Supplemental Table 1). The core 12-week multicomponent online intervention included two components: (i) a progressive weekly follow-along yoga, breathwork, and guided meditation video (20- to 30-min time commitment per video), which patients were asked to complete 2–3 times/week and (ii) a psychology-based video with accompanying activity informed by CBT or positive psychology (10- to 20-min time commitment per video), which patients were asked to complete weekly. Supplementary program videos included are as follows: a weekly introductory video (3–5 min) discussing the theme for the week and an IBD nutrition tip video (3–5 min). All programming was developed and delivered by qualified personnel. The program content varied from week to week and was distributed in a gated fashion with new content being ‘unlocked’ each week. The intervention was accessible *via* a password protected website and prior to launch, the content of the PPP had been reviewed and edited by our patient partners in informal focus groups.

Participants allocated to the PPP intervention had a baseline website orientation visit facilitated by a standardized training video. Intervention participants also received a 10-min telephone touchpoint once weekly from the research assistant. A standardized script probed about three areas on each weekly call: assistance required with the website, assistance required with the content, and participant feedback including adherence data to the core program. All intervention participants were asked to keep an adherence journal.

#### Data collection and outcome measures

At study baseline, data were gathered on patient characteristics including sex, age, smoking history, body mass index, hospitalization within 1 year of baseline, time since last flare, disease phenotype based on the Montreal classification,^36^ IBD medication, previous exposure to yoga and meditation, and baseline fecal calprotectin (FCP) levels. Primary and secondary outcome measures were collected at baseline and 12 weeks. The primary study outcome was the change in stress measured by the PSS. The PSS-10 is a 10-item scale that assesses the degree to which life has been experienced as unpredictable, uncontrollable, and overloaded over a 1-month time interval.^30^ Secondary outcomes included changes in the HBI for CD;^33^ the pMayo score for UC;^34^ the HADS – 14-item scale measuring possible anxiety and depressive states;^31^ Pittsburgh Sleep Quality Index – 19-item scale assessing sleep quality and disturbances over a 1-month interval;^37^ Short Inflammatory Bowel Disease Questionnaire (SIBDQ) – 10-item disease-specific HRQoL scale;^38^ EuroQol 5 Dimension and visual analog scale (EQ-5D-5L) – generic 5-item survey and visual analog scale to assess perceived health status;^39^ Connor–Davidson Resilience Scale (CD-RISC) – 25-item tool to assess resilience;^40^ and the Psychological Well-Being scale – 42-item survey to measure six aspects of well-being.^41^ Notably, patients with an ostomy or pouch did not have IBD disease severity (HBI or pMayo) recorded as these symptom indices are not validated in this patient population.

End-of-study acceptability/satisfaction questionnaires were completed by intervention participants to rate the intervention components on a 5-Point Likert scale ranging from ‘Very Dissatisfied’ to ‘Very Satisfied’. Using a 0–100 visual scale, the intervention allocated participants were asked about overall satisfaction and likelihood of continuing with the program and/or implementing discrete aspects of it after 12 weeks. Given the proposed relationship between stress and adverse outcomes, including inflammation,^8,10,11^ as part of our exploratory aims, for patients who were able to come into the study facility, baseline, and end-of-study blood samples were collected and analyzed for the following: high sensitivity C-reactive protein (hs-CRP), inflammatory cytokines [interleukin (IL)-6, IL-10], tumor necrosis factor-alpha (TNFα) and other proteins involved in growth and immunity (brain-derived neurotrophic factor (BDNF), indoleamine 2,3-dioxygenase (IDO), and triggering receptor expressed on myeloid cells 2 (TREM-2)). Analyses used the DuoSet enzyme-linked immunosorbent assay kits (R&D Biosystems, Minneapolis, MN, USA) following the manufacturer’s instructions. Serum dilutions required for this population and assay type were as follows: hs-CRP 10,000×; BDNF 400×; IDO 20×; TREM-2 4×; IL-10, IL-6, TNFα 1×.

#### Statistical analyses

The sample size calculation was based off of the PSS. A 20% reduction in a similar measure, the Perceived Stress Questionnaire has differentiated those with and without disease relapse.^42^ Based on our pilot data and the available literature,^43^ we estimated that only 5% of participants in the control group would have a 20% reduction in the PSS as compared to 30% in the intervention group. A sample size of 72 patients (36 control and 36 intervention) would have a power of 80% with an α of 0.05 to detect this difference. Allowing for a dropout rate of 30%, the sample size was increased to 100 patients.

Variables were reported as means and standard deviations or frequencies and proportions. Depending on the variable distribution, Pearson’s chi-squared, Fisher’s exact, or independent sample t-tests were used to compare characteristics between the intervention and control groups. To analyze the impact of the intervention on the PSS, we used analysis of covariance (ANCOVA). We tested the assumptions that there were no differences in baseline PSS values between groups and that the impact of the intervention did not differ depending on the baseline value. We conducted a linear regression model predicting change in PSS where the intervention was the primary predictor and PSS at the end of the study was the primary outcome. The model was adjusted for baseline PSS score and any additional baseline characteristics that were statistically different between the two groups. The beta coefficient of the intervention variable represented the absolute improvement of the intervention and relative improvement of the intervention was defined as the percentage difference in PSS score at the end of the study between the intervention and control group (absolute improvement ÷ mean end of study PSS score in control group). The same ANCOVA procedures were also used to test our secondary outcomes. The intention-to-treat analyses imputed missing values with the last observation carried forward method and were conducted using SAS software Version 9.4, Cary, NC, USA.

## Results

**Figure 1.**
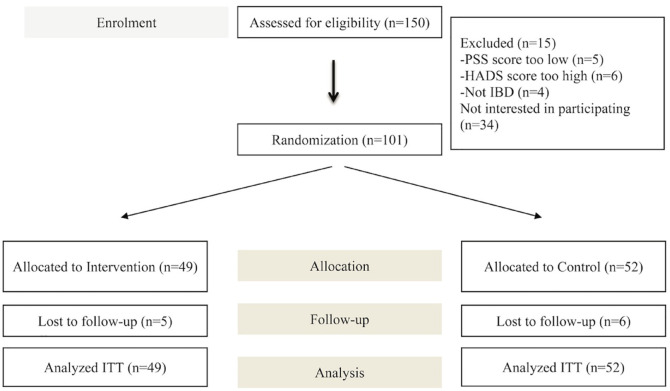
Patient recruitment and flow through the trial.

A total of 150 patients were screened for the trial: 34 declined to participate and 15 were excluded (Figure 1). Of the 101 patients randomized, 49 were allocated to the intervention group and 52 to the control group. After the 12-week intervention, the overall attrition was 10.9% (intervention *n* = 5; control *n* = 6) with 90 patients remaining at the end of study.

### Patient baseline characteristics

The mean age of participants was 42.5 ± 14.1 years, 75% were female, 48% had UC, and 52% had CD (Table 1). Within the previous year, 10% had been hospitalized for IBD-related reasons and 16.8% required a course of prednisone. Those randomized to the intervention group were older (45.4 ± 14.0 years *versus* 39.7 ± 13.8 years, *p* = 0.04) and had a greater proportion of patients with UC maintained on biologic therapy (66.7% *versus* 37.5%, *p* = 0.04). Participants had quiescent disease as indicated by the mean baseline measures of HBI of 4.2 ± 2.5, pMayo of 1.4 ± 1.6, CRP of 7.4 ± 26.5 mg/L, and FCP of 150.6 ± 372 mg/kg. Two patients with CD had an ileostomy and two patients with UC had an ileal pouch-anal anastomosis.

**Table 1.** Patient baseline characteristics.

	Total (*n* = 101)	Control group (*n* = 52)	Intervention group (*n* = 49)	*p* Value
IBD type				
UC	48 (47.5%)	24 (46.2%)	24 (49.0%)	0.84
CD	53 (52.4%)	28 (53.8%)	25 (51%)	
Age (years)	42.5 ± 14.1	39.7 ± 13.8	45.4 ± 14.0	*0.04*
Male sex	25 (24.7%)	12 (23.1%)	13 (26.5%)	0.69
Smoking history				
Smoker	3 (3.0%)	2 (3.8%)	1 (2.0%)	0.66
Ex-smoker	26 (25.7%)	13 (32.7%)	13 (26.5%)	
Nonsmoker	68 (67.3%)	33 (63.5%)	35 (71.4%)	
BMI	26.6 ± 5.4	27 ± 5.6	26.1 ± 5.1	0.41
IBD hospitalization within 1 year prior to baseline	10 (10%)	4 (7.7%)	6 (12.2%)	0.52
Time since last IBD flare (months)	31.4 ± 52.0	27.6 ± 28.1	35.6 ± 69.3	0.44
Clinical flare within 1 year prior to baseline	38 (37.6%)	19/52 (36.5%)	19/48 (39.6%)	0.84
Hemoglobin (g/L)	137.4 ± 19.1	136.1 ± 24.5	138.7 ± 12.0	0.52
CRP (mg/L)	7.4 ± 26.5	9.5 ± 34.6	5.6 ± 16.0	0.51
FCP (mg/kg)	150.6 ± 372	194.0 ± 538.6	121.4 ± 195.9	0.42
Previous exposure to yoga	36 (35.6%)	19 (36.5%)	17 (34.7%)	0.85
Previous exposure to meditation	25 (24.8%)	12 (23.1%)	13 (26.5%)	0.69
Self-reported history of depression	21 (20.8%)	11 (21.2%)	10 (20.4%)	1.0
Self-reported history of anxiety	39 (38.6%)	22 (42.3%)	17 (34.7%)	0.54
Self-reported history of PTSD	5 (5.0%)	2 (3.8%)	3 (6.1%)	0.6
CD symptoms, phenotype, and medications				
HBI	4.2 ± 2.5	4.5 ± 2.7	3.9 ± 2.4	0.42
Montreal classification				
L1	16/53 (30%)	9/28 (32%)	7/25 (28%)	0.38
L2	9/53 (17%)	4/28 (14%)	5/25 (20%)	
L3	22/53 (42%)	10/28 (36%)	12/25 (48%)	
L4	6/53 (11%)	5/28 (18%)	1/25 (4%)	
B1	30/53 (56.6%)	16/28 (57.1%)	14/25 (56%)	0.82
B2	18/53 (34.0%)	10/28 (35.7%)	8/25 (32%)	
B3	5/53 (9.4%)	2/28 (7.1%)	3/25 (12%)	
p	6/53 (11.3%)	3/28 (11%)	3/25 (12%)	0.63
5-ASA	11/53 (21%)	7/28 (25%)	4/25 (16%)	0.51
Azathioprine	10/53 (18.9%)	6/28 (21.4%)	4/25 (16%)	0.73
Biologics	32/53 (60.4%)	16/28 (57.1%)	16/25 (64%)	0.78
Prednisone within 1 year of baseline	6/53 (11.3%)	3/28 (10.7%)	3/25 (12%)	1.0
UC symptoms, phenotype, and medications				
pMayo score	1.4 ± 1.6	1.8 ± 1.8	± 1.3	0.17
Montreal classification				
E1	6/48 (12.5%)	2/24 (8.3%)	4/24 (16.7%)	0.62
E2	14/48 (29.2%)	8/24 (33.3%)	6/24 (25%)	
E3	28/48 (58.3%)	14/24 (58.3%)	14/24 (58.3%)	
5-ASA	22/48 (45.8%)	13/24 (54.2%)	9/24 (37.5%)	0.39
Azathioprine	8/24 (33.3%)	6/24 (25.0%)	2/24 (8.3%)	0.25
Biologics	25/48 (52.1%)	9/24 (37.5%)	16/24 (66.7%)	*0.04*
Prednisone within 1 year of baseline	11/48 (22.9%)	6/24 (25%)	6/24 (25%)	0.73

5-ASA, 5-aminosalicylate; BMI, body mass index; CD, Crohn’s disease; CRP, C-reactive protein; FCP, fecal calprotectin; HBI, Harvey-Bradshaw Index; IBD, inflammatory bowel disease; pMayo, partial Mayo; PTSD, post-traumatic stress disorder; UC, ulcerative colitis.

### Primary outcome

After adjusting for PSS, age, and biologic therapy at baseline in our linear regression model, there was a significant change in the PSS between baseline and end of study in the intervention arm compared to the control arm. This translated to an absolute improvement of 4.09 [95% confidence interval (CI), 1.67–5.83] and a relative improvement of 22.4% (95% CI, 10.5–34.3), *p* < 0.001 (Tables 2 and 3).

**Table 2.** Symptom indices, perceived stress, HRQoL, stress resilience, mental health, and sleep quality outcomes.

	Control group (*n* = 52)		Intervention group (*n* = 49)		*p* Value
	Baseline	End of study	Baseline	End of study	Between-group end of study
PSS	18.4 ± 6.0	18.2 ± 7.3	18.1 ± 5.8	14.3 ± 5.9	*<0.001*
pMayo	1.7 ± 1.8	1.3 ± 1.7	1.0 ± 1.3	0.8 ± 1.1	0.42
HBI	4.4 ± 2.7	4.5 ± 2.8	4.1 ± 2.3	4.2 ± 3.7	0.96
HADS total	14.1 ± 4.8	14.6 ± 5.4	14.1 ± 4.9	10.9 ± 4.2	*<0.001*
HADS anxiety	8.69 ± 3.25	8.96 ± 3.63	9.00 ± 3.39	7.00 ± 2.92	*<0.001*
HADS depression	5.20 ± 2.85	5.17 ± 3.10	4.98 ± 2.57	3.66 ± 2.18	*0.002*
Belief that stress impacts IBD	73.3 ± 22.7	72.0 ± 26.9	73.8 ± 14.3	68.7 ± 25.3	0.68
Global PSQI	8.2 ± 3.7	7.9 ± 3.6	8.6 ± 3.1	7.4 ± 3.5	0.187
SIBDQ	48.1 ± 8.8	50.2 ± 9.7	48.2 ± 8.9	55.2 ± 7.2	*<0.001*
EQ-5D visual analog scale	68.2 ± 18.5	70.4 ± 17.1	67.6 ± 15.0	77.2 ± 11.9	*0.003*
CD-RISC	67.7 ± 13.6	68.1 ± 14.8	64.5 ± 12.7	71.5 ± 12.8	*<0.001*
PWB self-acceptance	17.0 ± 3.5	17.3 ± 3.3	15.2 ± 3.5	16.7 ± 3.4	0.23
PWB purpose	17.0 ± 3.3	16.8 ± 3.5	15.6 ± 3.2	16.5 ± 3.3	0.31
PWB environmental mastery	14.9 ± 3.5	15.5 ± 3.5	14.2 ± 3.2	15.7 ± 3.5	0.24
PWB positive relations	16.4 ± 4.1	16.5 ± 4.0	15.8 ± 3.7	17.3 ± 3.6	*0.008*
PWB autonomy	15.8 ± 3.9	15.8 ± 3.8	15.1 ± 3.6	16.3 ± 3.0	0.082
PWB growth	18.3 ± 2.7	18.2 ± 3.2	17.3 ± 2.6	18.4 ± 2.6	0.124

CD-RISC, Connor–Davidson Resilience Scale; EQ-5D, EuroQol-5 Dimensions; HADS, Hospital Anxiety and Depression Scale; HBI, Harvey-Bradshaw Index; HRQoL, health-related quality of life; IBD, inflammatory bowel disease; pMayo, partial Mayo score; PSQI, Pittsburgh Sleep Quality Index; PSS, Perceived Stress Scale; PWB, Psychological Well-being Scale; SIBDQ, Short Inflammatory Bowel Disease Questionnaire. The italicized p-values are statistically significant.

**Table 3.** Change in symptom indices, stress, HRQoL, stress resilience, mental health, and sleep quality outcomes between the intervention and control groups.

	Absolute improvement (95% CI)	Relative improvement (%) (95% CI)	*p* Value
PSS	4.09 (1.92, 6.27)	22.4 (10.5, 34.3)	*<0.001*
pMayo	0.31 (−0.64, 1.27)	27.9 (−57.3, 113.0)	0.42
HBI	0.47 (−1.38, 2.32)	10.3 (−30.1, 50.8)	0.96
HADS total	3.83 (2.00, 5.65)	25.8 (13.5, 38.1)	*<0.001*
HADS anxiety	2.15 (1.04, 3.25)	23.7 (11.5, 35.9)	*<0.001*
HADS depression	1.55 (0.57, 2.54)	29.5 (10.9, 48.1)	*0.002*
Belief that stress impacts IBD	0.55 (−8.39, 9.50)	0.8 (−11.9, 13.5)	0.68
Global PSQI	0.67 (−0.29, 1.63)	8.5 (−3.7, 20.6)	0.187
SIBDQ	4.49 (2.03, 6.95)	8.9 (4.0, 13.7)	*<0.001*
EQ-5D visual analog scale	6.99 (2.30, 11.69)	9.9 (3.3, 16.6)	*0.003*
CD-RISC	7.01 (3.50, 10.52)	10.6 (5.3, 15.8)	*<0.001*
PWB self-acceptance	0.86 (−0.21, 1.93)	5.1 (−1.3, 11.6)	0.23
PWB purpose	0.82 (−0.31, 1.95)	5.0 (−1.9, 12.0)	0.31
PWB environmental mastery	0.89 (−0.27, 2.05)	5.9 (−1.7, 13.5)	0.24
PWB positive relations	1.30 (0.31, 2.28)	8.0 (1.9, 14.0)	*0.008*
PWB autonomy	0.91 (−0.17, 1.98)	5.8 (−1.1, 12.7)	0.082
PWB growth	0.82 (−0.25, 1.89)	4.6 (−1.4, 10.5)	0.124

CD-RISC, Connor–Davidson Resilience Scale; CI, confidence interval; EQ-5D, EuroQol-5 Dimensions; HADS, Hospital Anxiety and Depression Scale; HBI, Harvey-Bradshaw Index; HRQoL, health-related quality of life; IBD, inflammatory bowel disease; pMayo, partial Mayo score; PSQI, Pittsburgh Sleep Quality Index; PSS, Perceived Stress Scale; PWB, Psychological Well-being Scale; SIBDQ, Short Inflammatory Bowel Disease Questionnaire. The italicized p-values are statistically significant.

### Secondary outcomes

Data on secondary outcomes are presented in Tables 2 and 3. There was a significant absolute improvement in the HADS total (3.83, 95% CI: 2.00–5.65, *p* < 0.001), HADS anxiety (2.15; 95% CI: 1.04–3.25, *p* < 0.001), HADS depression (1.55, 95% CI: 0.57–2.54, *p* = 0.002), SIBDQ (4.49; 95% CI: 2.03–6.95, *p* < 0.001), EQ-5D-5L (6.99; 95% CI: 2.30–11.69, *p* = 0.003), and CD-RISC (7.01; 95% CI: 3.50–10.52, *p* < 0.001). Relative scores are presented in Table 3. There were no significant differences between study groups regarding HBI or pMayo scores (Table 3). Moreover, during the 3-month study period, 1/49 (2.0%) in the intervention arm *versus* 4/52 (7.7%) in the control arm experienced either a change/addition of biologic therapy or received a course of prednisone. This difference was not statistically different (*p* value = 0.36). Only one patient in the control arm underwent an IBD-related hospitalization and the same patient was also started on biologic therapy and prednisone.

### Exploratory outcomes

Baseline and end-of-study data for exploratory outcomes were available in 40 of the 49 intervention patients and 39 of the 52 control patients. There were no differences in baseline serum levels of TNFα, IL-6, IL-10, BDNF, TREM-2, and hs-CRP between groups. When converted to change from baseline to study end, only IDO significantly decreased in the intervention group (mean difference: −11.9; 95% CI: −22.7, −1.2, *p* = 0.03) (Supplemental Tables 2 and 3).

#### Adherence

Available in 44 of the 49 intervention patients who completed 12 weeks of programming, 91% met program adherence targets. Weekly program completion rates for the yoga, breathwork, and meditation video were as follows: once per week or less (4/44 or 9.1%), 2–3 times per week (29/44 or 65.9%), 4–5 times per week (6/44 or 13.6%), and daily (5/44 or 11.4%).

#### Program satisfaction (Figure 2)

Rated from 0 to 100 (‘Not Satisfied’ to ‘Extremely Satisfied’), the median satisfaction score was 89% with an interquartile range of 73–95%. Rated from 0 to 100 (‘Not Likely’ to ‘Extremely likely’), the median likelihood of continuing any element of the program after 12 weeks was 87% (interquartile range: 71–98%). Qualitative interview data added much depth to this survey data (reported separately).

**Figure 2.**
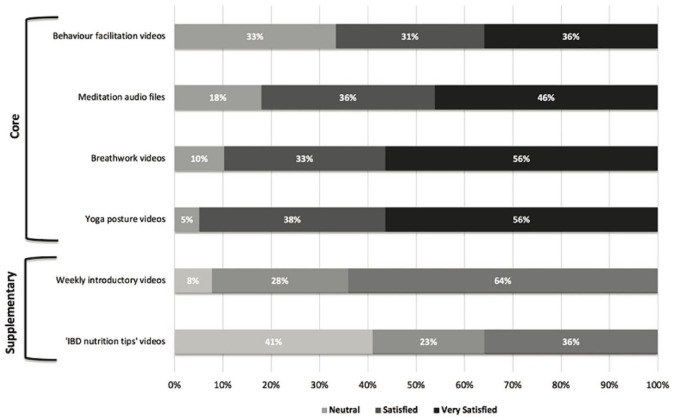
End-of-study participant satisfaction with the core and supplementary elements of the PPP program, scored using a 5-Point Likert Scale from very satisfied to very dissatisfied (*n* = 39). PPP, Peace Power Pack.

## Discussion

We report our findings of a multi-center RCT evaluating a 12-week multicomponent online stress reduction intervention in 101 participants with IBD. Statistically significant between-group reductions were seen in the primary outcome of stress, and secondary outcomes of anxiety and depression with improvements in the scores for quality of life and resilience, but no change in IBD disease severity or exploratory cytokine measures. The clinical significance of the findings on mental health can best be appreciated when looking at the HADS total score. This measure includes anxiety and depression. Data from several studies support a minimally clinically important difference of 1.5–2.^44,45^ The current study demonstrated an absolute improvement of 3.8 in this score, with a relative improvement of 26%. A similar relative improvement of 22% was seen in the PSS. High adherence and program acceptability were seen.

Our main findings are threefold. First, intervention efficacy was demonstrated across a range of mental health and HRQoL outcomes compared to controls. The IBD community has long acknowledged psychosocial health as a key clinical and research priority alongside mucosal healing.^46^ The current study adds to the existing literature not only because it had a significant effect on a range of outcomes, but also because these results were achievable by a scalable program that was delivered over a purely online platform, did not require direct psychologist support, and had high adherence and retention rates. The study was also unique in that it combined eastern practices of yoga and breathwork together with western practices of CBT and positive-psychology-based behavioral programming. Other published RCTs have evaluated online stress reduction/mind–body wellness interventions in IBD with varying durations and discordant results.^15,28,29^ A study published in 2016 provided IBD patients with a self-administered online CBT intervention. It was associated with increased HRQoL in those who completed it, but the attrition rate was 74% across 12 weeks of the study.^15^ Furthermore, in spite of similar participant baseline PSS-10 levels compared to our study, there were no reported differences in PSS-10 scores between the intervention and control groups, possibly related to the broader inclusion of participants with any baseline PSS-10 score. Mikocka-Walus *et al*. studied the effect of a 10-week online or face-to-face CBT intervention in IBD patients and demonstrated no difference in clinical remission at 24 months in spite of a potential benefit at 6 months.^29^ Their intervention completion rate was 25.7%. More recently, a RCT in 57 patients included four Internet-based therapy modules and four face-to-face support sessions carried out over 8 weeks. Survey-based measures were not assessed. The outcome assessment occurred after 6 months, demonstrating significant between-group differences in FCP and CRP levels.^28^ The durable effect of this intervention may be attributable to the mindfulness-based intervention itself and by extension, the 100% adherence rate of participants. Our RCT builds upon this literature as we report on both inflammatory biomarkers/cytokines and patient-reported outcomes.

Despite the significant impact on mental health and HRQoL measures, in the current study, there was no impact on IBD disease severity indices, inflammatory cytokines, or additional exploratory markers (e.g. BDNF, TREM-2). The decrease in IDO is interesting in that IDO is a rate-limiting enzyme in the kynurenine pathway, IDO promotes cell tolerance, and increases in IDO are associated with depression.^47,48^ Furthermore, IDO inhibition has been targeted for new therapeutic approaches in the treatment of depression and anxiety.^47^ The lack of changes in the inflammatory cytokines may have been anticipated as most participants were in clinical remission at baseline with well-controlled inflammation in the previous month (HBI of 4.2 ± 2.5, pMayo of 1.4 ± 1.6, CRP of 7.4 ± 26.5 mg/L where the upper limit of normal is 8.0 mg/L). The absence of an effect may also be attributable to the small sample size, and the relatively low frequency of the intervention, which required a time commitment of only 60–110 min per week.

Third, the study was associated with high rates of adherence and acceptability. As with all lifestyle-based health programs, adherence is an essential pre-requisite before seeing a desired impact on clinical outcomes.^49–52^ The standard 30-day retention rates of general unguided online wellness interventions are <5%^53^ which make the 91% adherence, 11% attrition, and 89% acceptability rates in the current study of particular interest. Of the published online mind–body/stress reduction interventions in IBD, attrition rates have ranged from 0% to 74%^15,27–29^ compared to 0% to 44% for in-person interventions.^17–24^ There are several potential learnings around adherence optimization that may be gleaned from the current study. First, the website (www.wellnesstoolbox.ca) was consistently reported to be easy-to-navigate despite the age range (18–74 years) of participants enrolled in the study. Second, the brief weekly telephone touchpoints were seen as an important mechanism to promote accountability. There is research to support the efficacy of such ‘brief’ touchpoints alongside online mental health interventions for anxiety and depression. Existing studies report similar efficacy and dropout rates regardless of whether a professionally qualified therapist or a ‘technician’ with basic training (as was the case in our study) provided the touchpoint.^54,55^ Third, the program adherence goals were achieved by 91% of participants who completed the 12-week programming – a signal that this was a reasonable adherence target that participants felt they could achieve. Fourth, the online delivery of the programming provided increased accessibility and flexibility to IBD participants with multiple competing obligations. Last, our program intervention materials were co-developed with guidance from our patient partners^56^ who provided valuable end-user input.

### Limitations and strengths

Consistent with other studies involving mind–body therapies (i.e. yoga, meditation),^57^ 75% of our participants were female making the results less generalizable to male patients. Although further evaluation is required, from the feedback we received, it is possible that alternative types of movement interventions (e.g. exercise based) may be more appealing to some male participants. Most intervention participants reported that they were ‘very likely’ to continue practicing elements of the program post-trial, but this was not formally re-evaluated. Disease relapses or flares during the study were not captured as we did not anticipate any significant changes during the short study period; however, there were changes in biologic therapy, prednisone use, and IBD-related hospitalizations. While some patients with clinical and biochemical disease activity were enrolled, <5% of participants underwent a change in biologic or prednisone therapy and <1% were hospitalized, reinforcing that the vast majority were not in the moderate-to-severe IBD category. The PPP intervention included multiple elements – (i) two core components: physical movement/mindfulness routine and the psychology informed behavioral skills content and (ii) optional supplementary content (a short weekly introductory video and nutrition tip video). Although on the surface this could be seen as too intensive for participants, it is important to note that our patient partners prioritized this variety during the study co-development as a means to support interest and adherence. Notably, for the core components alone, the minimum time commitment was 60–110 min per week. Based on feedback, the content will continue to be refined to meet participant needs. Lastly, adherence to the programming was self-reported by adherence logs and weekly check-ins, potentially over-estimating patient engagement. Study strengths include that limitations noted by other investigators were addressed: enrolling patients with higher levels of perceived stress, use of an online, multicomponent, self-directed intervention, and assessment of a broad panel of outcomes using validated measures.^58,59^

## Conclusions

This multicomponent intervention significantly improved perceived measures of stress, stress resiliency, psychiatric comorbidity, and HRQoL alongside high adherence and retention rates. Future studies can assess if different frequencies of the online intervention and different levels of support (brief check-ins *versus* no support) impact both patient-reported outcomes and objective IBD biomarkers. Additional work can also focus on the impact of these interventions in patients with active IBD and in preventing disease flares in those in remission. In addition, a longer follow-up duration of 3–6 months post-intervention would allow us to obtain insight into program continuation, IBD activity, and mental wellness.

## Supplemental Material

sj-doc-1-tag-10.1177_17562848221127238 – Supplemental material for A randomized controlled trial of a multicomponent online stress reduction intervention in inflammatory bowel disease Supplemental material, sj-doc-1-tag-10.1177_17562848221127238 for A randomized controlled trial of a multicomponent online stress reduction intervention in inflammatory bowel disease by Farhad Peerani, Makayla Watt, Kathleen P Ismond, Reid Whitlock, Lindsy Ambrosio, Naomi Hotte, Nicholas Mitchell, Robert J Bailey, Karen Kroeker, Levinus A Dieleman, Jesse Siffledeen, Allen Lim, Karen Wong, Brendan P Halloran, Daniel C Baumgart, Lorian Taylor, Maitreyi Raman, Karen L Madsen and Puneeta Tandon in Therapeutic Advances in Gastroenterology

sj-docx-2-tag-10.1177_17562848221127238 – Supplemental material for A randomized controlled trial of a multicomponent online stress reduction intervention in inflammatory bowel disease Supplemental material, sj-docx-2-tag-10.1177_17562848221127238 for A randomized controlled trial of a multicomponent online stress reduction intervention in inflammatory bowel disease by Farhad Peerani, Makayla Watt, Kathleen P Ismond, Reid Whitlock, Lindsy Ambrosio, Naomi Hotte, Nicholas Mitchell, Robert J Bailey, Karen Kroeker, Levinus A Dieleman, Jesse Siffledeen, Allen Lim, Karen Wong, Brendan P Halloran, Daniel C Baumgart, Lorian Taylor, Maitreyi Raman, Karen L Madsen and Puneeta Tandon in Therapeutic Advances in Gastroenterology

## Declarations

**Ethics approval and consent to participate:** Written informed consent was provided by participants. The UC and CD protocols were approved by the University of Alberta’s Research Ethics Office located in Edmonton, Alberta, Canada on 15 April 2018 (Pro00079377) and 26 June 2018 (Pro00082125), respectively. This trial was registered with www.ClinicalTrials.gov (Identifier NCT03831750).

**Consent for publication:** Not applicable.

**Availability of data and materials:** The data that support the findings of this study are available from the corresponding author upon reasonable request.
