# Supplementary material for: A randomized controlled trial of a multicomponent online stress reduction intervention in inflammatory bowel disease
Source: Ther Adv Gastroenterol. 2022 Sep 27;15:17562848221127238. doi: 10.1177/17562848221127238 (PMC9520184; doi:10.1177/17562848221127238)
Supplement: sj-docx-2-tag-10.1177_17562848221127238 – Supplemental material for A randomized controlled trial of a multicomponent online stress reduction intervention in inflammatory bowel disease [file sj-docx-2-tag-10.1177_17562848221127238.docx]

**Supplementary Table 1.** Description of the core online intervention

| **Week** | **Weekly intro, Behavior change and Meditation theme** | **Breathing Exercises Introduced** | **Yoga Postures Introduced** |
| --- | --- | --- | --- |
| **Week 1** | Connecting with your breath | Diaphragmatic breathing, alternate nostril breathing | Cat cow, yoga namaskar, tree, thunderbolt |
| **Week 2** | Connecting with your body |  |  |
| **Week 3** | Grounding | Sitali | Yogi squat, yoga namaskar in goddess |
| **Week 4** | Managing your thoughts |  | Triangle |
| **Week 5** | Personal power |  | Seated twist |
| **Week 6** | Social connectedness and gratitude |  | Happy baby, reclining twist, wind removing pose |
| **Week 7** | Self-expression |  | Beauty pose |
| **Week 8** | Letting go |  |  |
| **Week 9** | Forgiveness |  | Locust pose |
| **Week 10** | Visualization for health |  |  |
| **Week 11** | Visualization for other life goals |  |  |
| **Week 12** | End of program review – sustaining change |  |  |

**Supplementary Table 2.** Inflammatory bowel disease activity, inflammatory cytokines and stress biomarker outcomes

| **Variable** | **Control group (n=39)**  **Mean (95% CI)** | | **Intervention group (n=40)**  **(Mean (95% CI)** | | **Between Group p-values ^** | |
| --- | --- | --- | --- | --- | --- | --- |
|  | **Baseline** | **End of study** | **Baseline** | **End of study** | **Between Group**  **Baseline** | **Between Group**  **End of study** |
| hs-CRP (mg/L) | 6.7  (4.7, 8.7) | 5.5  (3.6, 7.4) | 5.2  (3.3, 7.0) | 5.1  (3.4, 6.8) | 0.29 | 0.79 |
| IL-6 (pg/mL) | 110.1  (49.5, 170.8) | 118.1  (55.7, 180.6) | 157.1  (85.4, 228.8) | 130.3  (65.5, 195.2) | 0.29 | 0.44 |
| IL-10 (pg/mL) | 486.9  (165.7, 809.1) | 504.3  (196.6, 812.1) | 435.9  (129.8, 741.9) | 385.5  (92.0, 679.1) | 0.56 | 0.75 |
| TNFα (pg/mL) | 295.1  (69.5, 520.7) | 293.9  (89.3, 498.5) | 434.6  (174.5, 694.6) | 353.3  (101.9, 604.7) | 0.20 | 0.66 |
| TREM-2 (ng/mL) | 6.4  (5.3, 7.6) | 6.4  (5.5, 7.2) | 5.7  (4.9, 6.6) | 6.0  (5.0, 7.1) | 0.28 | 0.33 |
| BDNF (ng/mL) | 44.0  (35.1, 52.8) | 43.3  (35.1, 51.5) | 74.5  (25.4, 123.5) | 40.5  (32.6, 48.3) | 0.14 | 0.50 |
| IDO (ng/mL) | 22.6  (1.4, 43.7) | 24.8  (3.8, 45.8) | 24.9  (6.4, 43.5) | 15.3  (1.7, 28.8) | 0.74 | 0.34 |

Abbreviations: hs-CRP – high sensitivity C-reactive protein; IL-6 – interleukin 6; IL-8 – interleukin 8; IL-10 – interleukin 10; TNFα – tumor necrosis factor-alpha; BDNF – Brain-derived neurotrophic factor; IDO – Indoleamine 2,3-dioxygenase. ^ Displayed p-values are cross-sectional for each discrete time point (Fisher’s exact t-test).

**Supplementary Table 3.** Change in inflammatory bowel disease activity, inflammatory cytokines and stress biomarker outcomes between the control and intervention groups

| **Variable** | **Control Change ^ (n=39)** | **Intervention Change ^ (n=40)** | **Difference between means** | **Between group change p-value*** | **Absolute improvement**  **(95% CI)** | **Relative improvement (%)**  **(95% CI)** |  |
| --- | --- | --- | --- | --- | --- | --- | --- |
| hs-CRP (mg/L) | -1.2 (-3.3, 0.8) | -0.1 (-1.9, 1.6) | 1.1 (-1.6, 3.8) | 0.41 | 0.0 (-2.3, 2.4) | 0.9 (-43.5, 45.3) |  |
| IL-6 (pg/mL) | 8.0 (-20.1, 36.1) | -26.8 (-66.1, 12.5) | -34.8 (-82.5, 12.9) | 0.15 | 28.5 (-20.0, 76.9) | 21.2 (-14.9, 57.4) |  |
| IL-10 (pg/mL) | 17.4 (-192.7, 227.6) | -50.3 (-190.3, 89.7) | -67.8 (-316.8, 181.3) | 0.59 | 103.5 (-142.5, 349.6) | 21.6 (-29.7, 72.8) |  |
| TNFα (pg/mL) | -1.2 (-95.5, 93.1) | -81.3 (-180.0, 17.5) | -80.1 (-214.5, 54.3) | 0.24 | 57.3 (-78.8, 193.4) | 16.3 (-22.4, 55.0) |  |
| TREM-2 (ng/mL) | -0.1 (-1.0, 0.8) | 0.3 (-0.8, 1.4) | 0.4 (-1.1 to 1.8) | 0.62 | 1.0 (-0.1, 2.1) | 14.9 (-2.1, 32.0) |  |
| BDNF (ng/mL) | -0.7 (-11.1, 9.7) | -34.0 (-82.6, 14.6) | -33.28 (-82.9, 16.3) | 0.18 | 3.1 (-8.5, 14.8) | 11.6 (-31.4, 54.7) |  |
| IDO (ng/mL) | 2.2 (-1.1, 5.6) | -9.7 (-19.7, 0.6) | -11.9 (-22.7, -1.2) | *0.030* | 13.1 (2.8, 23.3) | 48.4 (10.5, 86.3) |  |

Abbreviations: hs-CRP – high sensitivity C-reactive protein; IL-6 – interleukin 6; IL-8 – interleukin 8; IL-10 – interleukin 10; TNFα – tumor necrosis factor-alpha; BDNF – Brain-derived neurotrophic factor; IDO – Indoleamine 2,3-dioxygenase. ^ Concentration change from baseline to 12 weeks, subtracted from endpoint values with-in each person, prior to group average and 95% confidence level calculations. * Welch’s t-test displayed.
